# Genetic Characterization of *Streptococcus pyogenes emm*89 Strains Isolated in Japan From 2011 to 2019

**DOI:** 10.1097/IM9.0000000000000038

**Published:** 2020-10-21

**Authors:** Yujiro Hirose, Masaya Yamaguchi, Norihiko Takemoto, Tohru Miyoshi-Akiyama, Tomoko Sumitomo, Masanobu Nakata, Tadayoshi Ikebe, Tomoki Hanada, Takahiro Yamaguchi, Ryuji Kawahara, Rumi Okuno, Hitoshi Otsuka, Yuko Matsumoto, Yuji Terashima, Yu Kazawa, Noriko Nakanishi, Kaoru Uchida, Yumi Akiyama, Kaori Iwabuchi, Chikara Nakagawa, Kazunari Yamamoto, Victor Nizet, Shigetada Kawabata

**Affiliations:** 1 Department of Oral and Molecular Microbiology, Osaka University Graduate School of Dentistry, Suita, Osaka, Japan; 2 Department of Pediatrics, University of California at San Diego School of Medicine, La Jolla, CA, USA; 3 Department of Infectious Diseases, Research Institute, National Center for Global Health and Medicine, Tokyo, Japan; 4 Department of Oral Microbiology, Kagoshima University Graduate School of Medical and Dental Sciences, Kagoshima, Japan; 5 Department of Bacteriology I, National Institute of Infectious Diseases, Tokyo, Japan; 6 Division of Microbiology, Osaka Institute of Public Health, Osaka City, Osaka, Japan; 7 Department of Microbiology, Tokyo Metropolitan Institute of Public Health, Tokyo, Japan; 8 Department of Public Health Sciences, Yamaguchi Prefectural Institute of Public Health and Environment Yamaguchi City, Yamaguchi, Japan; 9 Microbiological Testing and Research Division, Yokohama City Institute of Public Health, Yokohama, Kanagawa, Japan; 10 Department of Microbiology, Fukushima Prefectural Institute of Public Health, Fukushima City, Fukushima, Japan; 11 Department of Infectious Diseases, Kobe Institute of Health, Kobe, Hyogo, Japan; 12 Department of Bacteriology, Toyama Institute of Health, Imizu, Toyama, Japan; 13 Infectious Disease Research Division, Hyogo Prefectural Institute of Public Health Science, Kakogawa, Hyogo, Japan; 14 Department of Health Science, Iwate Prefectural Research Institute for Environmental Sciences and Public Health, Morioka, Iwate, Japan; 15 Division of Microbiology, Kyoto City Institute of Health and Environmental Sciences, Kyoto City, Kyoto, Japan; 16 Niigata City Institute of Public Health and the Environment, Niigata City, Niigata, Japan; 17 Skaggs School of Pharmaceutical Sciences, University of California at San Diego, La Jolla, CA, USA.

**Keywords:** clade 3, *emm*89, streptococcal toxic shock syndrome, *Streptococcus pyogenes*

## Abstract

Invasive infection caused by *Streptococcus pyogenes emm*89 strains has been increasing in several countries linked to a recently emergent clade of *emm*89 strains, designated clade 3. In Japan, the features of *emm*89 *S. pyogenes* strains, such as clade classification, remains unknown. In this study, we collected *emm*89 strains isolated from both streptococcal toxic shock syndrome (STSS) (89 STSS isolates) and noninvasive infections (72 non-STSS isolates) in Japan from 2011 to 2019, and conducted whole-genome sequencing and comparative analysis, which resulted in classification of a large majority into clade 3 regardless of disease severity. In addition, invasive disease-associated factors were found among *emm*89 strains, including mutations of control of virulence sensor, and absence of the *hylP1* gene encoding hyaluronidase. These findings provide new insights into genetic features of *emm*89 strains.

## Introduction

*Streptococcus pyogenes* is a human-specific pathogen known to cause a broad spectrum of diseases ranging from mild throat and skin infections to life-threatening invasive diseases.^1,2^ Worldwide, it has been estimated that there are over 111 million cases of streptococcal pyoderma and 616 million cases of *S. pyogenes* pharyngitis, with 663,000 cases of invasive infection each year.^3^ The most severe manifestation noted is streptococcal toxic shock syndrome (STSS), which results in significant mortality with reported incidence rates ranging from 23% to 81%.^4^ Current estimates suggest that the incidence of STSS is increasing throughout the world,^4,5^ thus genetic characterization of recently emerged strains is useful for investigating novel therapeutic targets.

The M protein is a surface protein and the best studied of *S. pyogenes* virulence factors.^6^ Although *S. pyogenes* typing has been historically conducted on the basis of M protein antigenicity, sequence typing of the *emm* region encoding the hyper-variable region of the M protein has also been widely applied as another method and used to classify the organism into at least 240 *emm* sequence types.^7,8^ In recent years, an increased incidence of invasive infection caused by *emm*89 *S. pyogenes* strains has been reported in both Europe and North America,^9–11^ with genome sequence analysis findings of *emm*89 strains used to identify 3 major genetically distinct strain clusters, designated as clade 1, 2, and 3. Importantly, concurrent with the period in the early 2000s that featured a significant increase in number and frequency of invasive *emm*89 infections, clade 3 strains emerged and showed rapid expansion.^9,12^

Clade 3 strains are characterized by absence of the *hasABC* locus, which is responsible for synthesis of the hyaluronic acid capsule, as well as the presence of the *nga-ifs-slo* locus variant, associated with increased expressions of NADase and cytolysin.^10,12^ Those reports indicated that most isolates from patients with invasive diseases can be classified into clade 3. In Japan, the incidence of STSS caused by *emm*89 *S. pyogenes* is increasing,^13^ though the features of *emm*89 *S. pyogenes* strains, such as clade classification, remain unknown.

In this study, we examined *emm*89 *S. pyogenes* of both isolates from patients with STSS (STSS isolates) and isolates from non-invasive infections (non-STSS isolates) isolated from 2011 to 2019 in Japan. Our results showed most of the *emm*89 *S. pyogenes* isolated in Japan were classified as clade 3. In addition, we found some factors were associated with invasive infections isolates among the *emm*89 strains. These findings support the understanding of the recently emerged *emm*89 *S. pyogenes*.

## Results

### High-isolation rates of *emm*89 *S. pyogenes* clade 3

**Figure 1:**
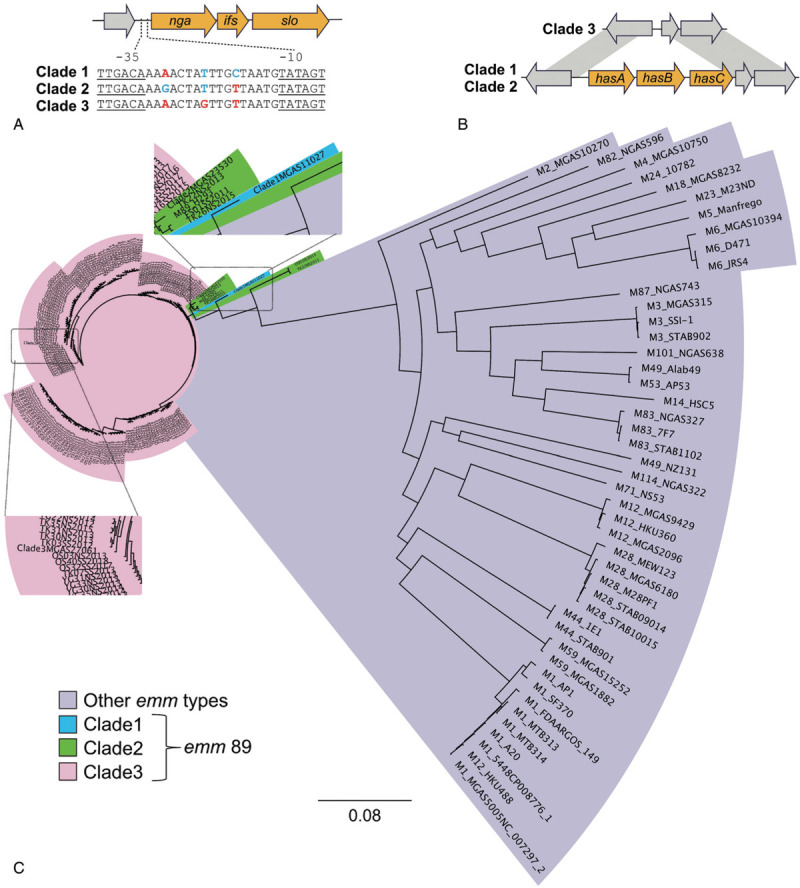
**Classification criteria for clade, and genetic relationships among various *S. pyogenes* strains**. A. Schematic diagram showing *nga* promoter region sequences for each clade. B. Schematic alignment of *hasABC* operon for clade 1, 2, and 3. *hasABC* was not found in clade 3. C. Genetic relationships between *emm*89 strains in Japan and various *emm*/M protein serotype strains. Genetic relationships were inferred among 49 GAS strains of 23 *emm*/M types based on 73,030 concatenated core chromosomal SNPs using the maximum likelihood method. Three closed genomes (clade 1, MGAS11027; clade 2, MGAS23530; clade 3, MGAS27061) were used as references. *S. pyogenes emm*89 strain clade 1, 2, and 3 are shaded in blue, green, and pink, respectively. Various *emm*/M protein serotypes except *emm*89 strains are shaded in purple. 20xx indicates year of isolation. Scale bar indicates nucleotide substitutions per site. FS: regions containing Sapporo City, Iwate Prefecture, Fukushima Prefecture, Sendai City, and Niigata City; TY; Toyama Prefecture; TK: Tokyo Prefecture; YH: Yokohama City; OS: regions containing Shiga Prefecture, Kyoto City, Osaka Prefecture, and Hyogo Prefecture; YG; Yamaguchi Prefecture; SS; isolates from STSS patients; NS: pharyngeal or asymptomatic isolates.

An important feature for characterization of clade 1, 2, and 3 is the *nga* promoter region sequence (Figure 1A). That sequence of *emm*89 clade 3 strains is identical to the *nga* promoter region present in pandemic *emm*1 strains.^12^ Furthermore, the *nga* promoter region sequence of clade 3 strains is associated with elevated production of *S. pyogenes* NADase and streptolysin O, secreted cytolytic toxins that contribute to its invasive phenotype.^12,14^ Clade 3 *emm*89 strains also lack the *hasABC* region required for synthesis of the hyaluronic acid capsule (Figure 1B). We classified all *emm*89 strains into clade 1, 2, or 3 based on both the *nga* promoter region sequence and presence of *hasABC* genes. Regardless of disease severity, nearly all (156 of 161 isolates in this study, 96.9%) of the strains were determined to be clade 3 (Supplementary Dataset 1), while only 5 showed characteristics of clade 2 strains and no clade 1 strain was detected. Chi-square test results suggested that clade 3 is not associated with invasive infections (Table 1), although the powering of the test was low due to small sample size.

**Table 1:** Factors associated with invasive infections

Factor	RefSeq locus tag	Gene symbol	STSS (n = 89)	Non-STSS (n = 72)	*χ*^2^ value^∗^	*P*-value	Power of test
Associated with STSS							
Mutation in CovS	MGAS23530_0304	*covS*	20	2	13.09	2.98.E-04	0.95
Absence of *hylP1* gene	MGAS27061_0574	*hylP1*	34	10	11.85	5.78.E-04	0.93
Not associated with STSS							
Clade 3			87	69	0.49	4.85.E-01	0.11
Mutation in CovR	MGAS23530_0303	*covR*	4	0	3.32	6.85.E-02	0.45
Mutation in Rgg	MGAS23530_1555	*rgg*	3	4	0.46	4.99.E-01	0.10

CovS: control of virulence sensor; STSS: streptococcal toxic shock syndrome.

∗ *χ*^2^ values for evaluating association between STSS and factors.

### Phylogenetic relationships of *emm*89 *S. pyogenes* based on the core single nucleotide polymorphisms (SNPs)

**Figure 2:**
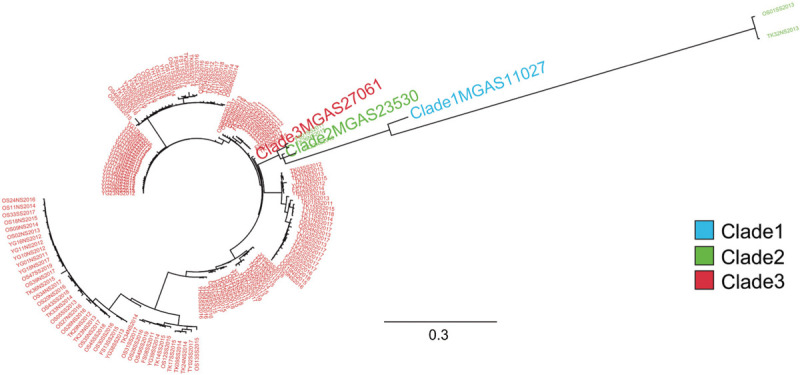
**Genetic relationships among *emm*89 strains in Japan and their clades**. Genetic relationships between *emm*89 strains in Japan and *emm*89 reference strains (clade 1, MGAS11027; clade 2, MGAS23530; clade 3, MGAS27061) are shown. Genetic relationships were inferred among 164 strains based on 8866 concatenated core chromosomal SNPs using the maximum likelihood method. Strains are colored based on clade, as indicated in the index. Scale bar indicates nucleotide substitutions per site.

We constructed kSNP trees using 161 *emm*89 assembled contigs and 49 other *S. pyogenes* complete genome sequences (Figure 1C) (for information regarding strains used in this study see Supplementary Dataset 1 and 2). As control genomes of the *emm*89 strains, 3 reference genomes from representative pre-epidemic (clade 1, MGAS11027; clade 2, MGAS23530) and epidemic (clade 3, MGAS27061) *emm*89 strains sequenced in a previous study by Zhu *et al.* were used.^15^ We also constructed kSNP trees using assembled contigs of the 161 *emm*89 strains and three control genomes (Figure 2). The phylogenetic tree based on the core SNPs showed that 156 *emm*89 clade 3 strains and three *emm*89 clade 2 strains indicated genetically close relationships with the reference genome of clade 3 (MGAS27061) and clade 2 (MGAS23530), respectively. These 159 *emm*89 *S. pyogenes* strains were consistent with the results of classification of clades based on both the *nga* promoter region sequences and presence of the *hasABC* genes (Supplementary Dataset 1). OS01SS2013 and TK32NS2013, which were classified to clade 2 based on the sequences of *nga* promoter region and presence of the *hasABC* genes, indicated genetically distant from the other *emm*89 isolates or the reference genome of clade 1 (MGAS11027). However, we suspect these two strains have not arisen through *emm*-type switching, because the two strains showed closer genetic relationship to other 159 *emm*89 strains than other *S. pyogenes* strains of 23* emm*/M types as branch length indicated. It was difficult to follow the branching relationship of *S. pyogenes emm*89 strains (Figure 1C and Figure 2). Therefore, we drew a cladogram to more clearly show the bootstrap values and branching relationships among the strains (Supplementary Figures 1 and 2).

### Invasive disease-associated factors

We tried to find invasive disease-associated mutations using SNPs (Table 2). Among the virulence factors, an amino acid substitution of streptokinase (Ska, Isoleucine to threonine substitution at position 17: I17T) was associated with invasive disease, while amino acid substitutions of streptolysin S biosynthesis protein C (SagC, Proline to serine substitution at position 223: P223S) were associated with noninvasive infections.

**Table 2:** Invasive disease-associated mutations

RefSeq number	Gene symbol	Product	Protein effect	STSS (n = 89)	Non-STSS (n = 72)	*χ*^2^ value^∗^	*P*-value
0566	*sagC*	Streptolysin S biosynthesis protein C	P223S	2	9	6.57	1.00E-02
1504	*ska*	Streptokinase	I17T	28	3	19.07	1.00E-05

STSS: streptococcal toxic shock syndrome.

∗ *χ*^2^ values for evaluating association between STSS and protein effect.

Mutations of the CovRS virulence regulator derepress virulence genes, leading to a hypervirulent phenotype of *S. pyogenes*.^16,17^ Of the 161 clinical isolates of *emm*89 *S. pyogenes* identified in Japan, 20 STSS isolates (22.5%) and 2 non-STSS isolates (2.7%) showed control of virulence sensor (CovS) mutations (*P* = 0.000298, *χ*^2^ analysis) (Table 1, Supplementary Dataset 1). On the other hand, the frequency of CovR mutations in the isolates was not associated with invasive infections. Although the Rgg mutation has also been reported as an important factor in the pathogenesis of invasive infections,^18^ that was not found to be relevant to invasive infections in this study. It appears that insufficient detection of these mutations led to the low power in the test, and explains the discrepancies with past reports.

**Figure 3:**
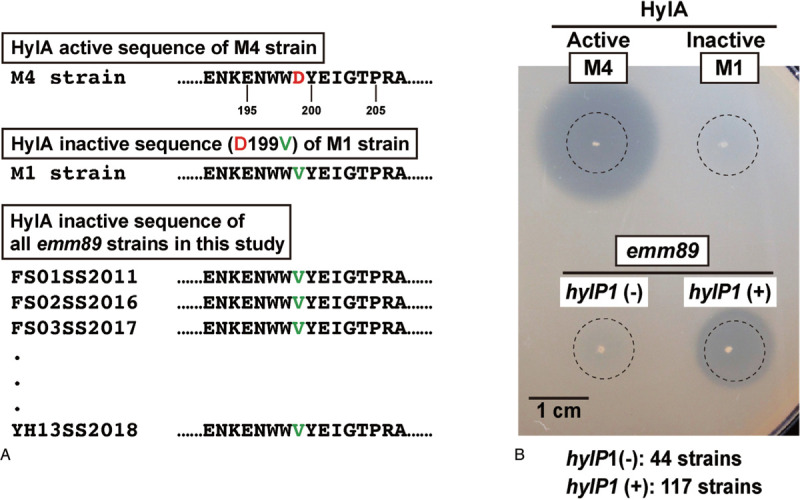
**Hyaluronidase activity of *emm*89 *S. pyogenes***. A. Hyaluronidase HylA active or inactive sequence. Serotype M4 strain was shown to harbor HylA activity and serotype M1 strain possession of Asp to Val substitution at position 199 of the HylA sequence (D199V), which abolishes hyaluronidase activity.^20^ B. Hyaluronidase activity on hyaluronan agar plate. Dotted circles indicate colony locations before incubating with 2 N acetic acid solution. Although 161 *emm*89 strains in this study harbored D199V in the HylA sequence, 117 of the strains were indicated to have hyaluronidase activity.

Hyaluronidase secreted by Group B *Streptococcus* cleaves pro-inflammatory hyaluronan, which contributes to evasion of host immunity.^19^ Accordingly, we screened the 161 isolated *emm*89 strains for hyaluronidase activity to investigate their involvement in invasive infections. Serotype M4 strain 4063-05 was used as a positive control for hyaluronidase (HylA) activity, while the negative control was serotype M1 strain 5448, which possesses an aspartic acid to valine substitution at position 199 of the HylA sequence (D199V) that abolishes hyaluronidase activity.^20^ Although all 161 strains harbored D199V in the HylA sequence, 117 showed hyaluronidase activity when cultured on hyaluronan agar plates (Figure 3), and that activity was consistent with the presence of the *hylP1* gene (Supplementary Dataset 1). The *hylP1* gene was absent in 34 (38.2%) of the STSS isolates and 10 (12.7%) of the non-STSS isolates (*P* = 0.00578, *χ*^2^ analysis) (Table 1). HylP1 is one of the key bacteriophage-encoded virulence factors.^21^ However, in regards to S*. pyogenes emm*89, absence of *hylP1* gene is relevant to invasive infections.

To reflect all regions as a confounding factor, we attempted to conduct Cochran–Mantel–Haenszel (CMH) test on invasive disease-associated factors. However, our research collection does not contain non-STSS isolates from the FS, TY, or YH regions. In addition, CovS mutations in isolates from the YG region, amino acid substitutions of SagC (P223S) in isolates from the TK region, and amino acid substitutions of Ska (I17T) in isolates from the YG region were not detected. Therefore, CMH tests were conducted using those areas available for appropriate analysis (Supplementary Table 1).

## Discussion

The emergence of clade 3 strains has already been reported in Europe, including Finland, Iceland, England, and Portugal, as well as North America (United States and Canada).^9–11,22^ These reports suggest that recently documented invasive infections caused by *emm*89 strains are due to the expansion of clade 3 as a dominant clade. Our results also indicate that most *emm*89 strains isolated in Japan since 2011 are members of the recently emerged genetic clade 3. Although a control group of non-STSS invasive isolates was not included in the study, we found possible factors associated with STSS among *emm*89 strains.

Streptokinase (Ska) is highly specific for human plasminogen and contributes to streptococcal virulence by generating plasmin, which leads to bacterial spread from a primary focus of infection by causing fibrinolysis, as well as degradation of the extracellular matrix and basement membrane components.^23^ The isoleucine or threonine residues at position 17 of the Ska sequence of *emm*89 strains are located in the streptokinase α domain. The α domain contributes to interactions between the catalytic domain of human plasminogen and streptokinase.^24^ Although proline to serine substitution at position 223 of the SagC sequence (P223S) is detected as non-invasive disease-associated mutations in this study, and there are no studies reported regarding the relationship between these mutations and the activity of the encoded enzyme. The limitation of our study is the lack of a robust activity assay to prove whether detected mutations affected the overall virulence of the pathogen. In addition, a further limitation of this study is the modest total number of *S. pyogenes* strains and the geographic bias in regions from where the strains were collected. Further verification and a larger sample size will ultimately be needed to confirm the relevance between our findings and their detailed contribution to bacterial pathogenesis.

Ikebe *et al*. reported that CovRS and Rgg mutations have important roles in the pathogenesis of STSS.^18^ The present results showed mutations of CovS associated with invasive infections, while those of CovR and Rgg did not have such an association. However, it is possible that relevance could not be determined due to the small number of strains examined. Two CovS mutations detected in this study were previously found among isolates from STSS patients.^18^ Truncating mutations at amino acid 35 or 39 (10 of 89 STSS isolates in this study) were previously detected in various *emm* type of STSS isolates (18 of 164 STSS isolates), while these mutations were not detected in non-STSS isolates from our and previous studies. These findings suggest that some particular SNPs affect the overall pathogenicity of the bacteria directly, while many additional SNPs may contribute to pathogenicity in cooperation with other factors. Further investigation will be required to reveal whether invasive disease-associated SNPs in this study function alone or not.

*emm*89 clade 3 strains lack hyaluronic acid capsule synthesis genes (*hasABC*), as previously reported for the *S. pyogenes* M4 and M22 strains.^25^ M4 and M22 are the only *S. pyogenes* strains known to possess an active HylA sequence,^20^ whereas that was not found in the *emm*89 strains examined in the present study. Therefore, a genomic type of *emm*89 clade 3 strain harboring an inactive HylA sequence, even though *hasABC* is deleted, has yet to be reported. We also found no association of invasive infections with HylP1-inactive *emm*89 strains. It was previously reported that an HylA-deficient mutant of *S. pyogenes* M4 did not display a significant reduction in virulence as compared with a wild-type strain.^26^ Together, these findings suggest that hyaluronidase activity may be dispensable for invasive infection by a specific subset of *S. pyogenes* strains.

*S. pyogenes emm*89 clade 3 strains have recently emerged in Japan. Invasive disease-associated factors were found among *emm*89 strains, including mutations of CovS, and absence of the *hylP1* gene encoding hyaluronidase. However, total number of isolates in our study may not be enough for statistical analysis. In addition, invasive disease-associated factors we found were not verified by activity assays. Therefore, those involvement in pathogenicity is unclear. We hope that our paper could be a source of information for future larger-scale analysis and comparisons with the genetic characteristics of strains from other countries.

## Materials and methods

### Bacterial isolates

Clinical isolates were sent from multiple medical institutions to public health institutions. The diagnostic criteria used for determining *S. pyogenes*-induced STSS were based on definitive cases described by the Working Group on Severe Streptococcal Infections (1993).^27^ Briefly, STSS isolates used in this study were isolated from normally sterile site. In addition, patients of STSS showed more than three clinical signs of severity, including hypotension, and more than two of renal impairment, coagulopathy, liver involvement, adult respiratory distress syndrome, a generalized erythematous macular rash, or soft-tissue necrosis. Non-STSS isolates in this study were isolated from pharyngitis, tonsillitis, or superficial skin lesion. The *emm* gene sequencing was performed as previously described by Beall *et al*.,^28^ with modifications as described at the Centers for Disease and Control website (https://www.cdc.gov/streplab/groupa-strep/emm-background.html).

Some isolates used in this study were previously characterized, which investigated different points as compared to our research. The Working Group for *β*-Hemolytic Streptococci in Japan has determined T serotypes for 75 strains of STSS isolates (from 2011 to 2017) used in this study.^29^ In addition, five strains of STSS isolates (from 2011 to 2012) used in this study were contained in the report for *emm* distribution in Japan (Supplementary Dataset 1).^13^

In Japan from 2011 to 2019, the number of STSS cases caused by *S. pyogenes* was 3857, among which *emm* typing was conducted in 1897 cases (49.2% of 3857 cases). The number of *emm*89 strain was detected in 183 cases (9.6% of 1897cases) and we could collect 89 strains (48.6% of 183 cases). Therefore, we estimate 23.9% (49.2 × 48.6 × 100) of Japanese *emm*89 strains which caused STSS were used in this study. The number of non-STSS isolates collected in this study is estimated about ∼0.3% of the total number of incident cases reported, because the number of cases of pharyngitis is reported around 300,000 per year in Japan. The discrepancy of these numbers was caused based on the differences of the accessibility to multiple medical institutions or public health institutions.

### Ethical statement

The study protocol including use of human subjects was approved by the Ethics Committee of Osaka University Graduate School of Dentistry (Approval No: H29-E16-1). All procedures were performed in accordance with both ethical standards for human experimentation and the latest revised version of the Declaration of Helsinki. We obtained clinical isolates of *S. pyogenes* retrospectively, and we utilized opt-out consent procedure instead of obtaining written informed consent from the patients. Therefore, we planned not to receive patient disease information, and our plan was judged to be ethical by the Ethics Committee.

### Genomic DNA sequencing

Isolates were cultured overnight in screw-capped glass tubes (Pyrex, Iwaki Glass, Tokyo, Japan) filled with Todd-Hewitt broth (BD Biosciences, San Jose, CA, USA) and supplemented with 0.2% yeast extract (BD Biosciences) (THY) at 37°C in an ambient atmosphere. Bacterial cells were lysed with 10 units/mL mutanolysin (Sigma, St. Louis, MO, USA), 10 mg/mL lysozyme (Wako, Osaka, Japan), and 0.5 mg/mL achromopeptidase (Wako), and genomic DNA was extracted from overnight cell cultures using a Maxwell^®^ RSC instrument with a Maxwell^®^ RSC PureFood GMO and Authentication Kit (Promega Corporation, Madison, WI, USA). Paired-end libraries were generated from extracted DNA with a Nextera^®^ XT DNA kit (Illumina, San Diego, CA, USA). Libraries were sequenced with Illumina instruments (HiSeq X and Miseq) and paired-end sequence reads (150 bp and 301 bp, respectively) were obtained.

### Data preprocessing and phylogenetic tree visualization

For subsequent genetic analyses, obtained sequence data were preprocessed to remove adapters and low-quality sequences with BBduk (https://github.com/BioInfoTools/BBMap). Next, obtained data were assembled de novo using a Unicycler (https://github.com/rrwick/Unicycler). Assembled contig and complete genome sequences (Supplementary Dataset 2) were analyzed using the kSNP3 program, version 3.1.1,^30^ which is able to generate phylogenetic trees based on the core SNPs by analyzing both complete genomes and unfinished genomes in assembled contigs. The Kchooser program in kSNP3 was used to estimate the optimum k-mer values. Maximum likelihood trees were visualized using the Geneious Prime 2019.2 software package (Biomatters, Auckland, New Zealand).

### Characterization of the assembled contig

To classify the clade of *emm*89 strains as described previously,^15^ the sequences of the *hasABC* or the *nga* promoter regions were pulled from the genome of MGAS23530, an *emm*89 clade 2 reference strain. The sequences of the *hylA* or the *hylP1* were pulled from the genome of MGAS10750, an M4 reference strain. Pulled sequences were compared with assembled contig of each strain using nucleotide BLAST (https://blast.ncbi.nlm.nih.gov/Blast.cgi).

### Detection of SNPs

Preprocessed reads were also aligned to the genome of MGAS23530, an *emm*89 clade 2 reference strain, using the Bowtie2 package (http://bowtie-bio.sourceforge.net/bowtie2/index.shtml). The genome of MGAS23530 was previously used in analysis of *emm*89 strains.^9,22^. Since the genome of MGAS23530 lacks prophages, the resultant polymorphism calls were made relative to only the core chromosome. SNPs identified relative to the 1,709,394 nucleotides of MGAS23530 were concatenated and visualized using Geneious Prime 2019.2. A Chi-square tests, power estimations, and CMH tests were conducted using Exploratory Public (Exploratory, Inc., San Francisco, CA, USA) or BellCurve for Excel (Social Survey Research Information Co., Ltd., Tokyo, Japan).

### Screening of bacterial strains for hyaluronidase activity

After culturing overnight, 10 μL of *S. pyogenes* was inoculated onto the surface of a hyaluronan agar plate containing Todd-Hewitt broth (BD Biosciences), 1% yeast extract (BD Biosciences), 400 μg/mL hyaluronic acid sodium salt (Wako), 1% bovine serum albumin (Sigma), and 2% Noble agar (BD Difco, Franklin Lakes, NJ, USA). Following overnight incubation, agar plates were flooded with 2 N acetic acid solution and further incubated for 15 minutes. Strains exhibiting a halo zone around the colony were deemed positive for hyaluronidase activity. *S. pyogenes* strain 5448 (serotype M1, negative for hyaluronidase activity) and strain 4063-05 (serotype M4, positive for hyaluronidase activity) were employed as positive and negative controls, respectively.^20^

### Data access

Data for the 161 sequenced *emm*89 genomes were deposited into the DDBJ sequence read archive under accession number DRA009110.

## Supplementary Material

Supplemental Digital Content
