# Supplementary material for: Genetic Characterization of Streptococcus pyogenes emm89 Strains Isolated in Japan From 2011 to 2019
Source: Infect Microbes Dis. 2020 Oct 21;2(4):160–6. doi: 10.1097/IM9.0000000000000038 (PMC7769053; doi:10.1097/IM9.0000000000000038)
Supplement: Supplemental Digital Content [file im9-2-160-s001.pdf]

Supplemental digital content (SDC)

Genetic characterization of *Streptococcus pyogenes emm*89 strains isolated in Japan from 2011 to 2019

Yujiro Hirose[1,2,*], Masaya Yamaguchi[1], Norihiko Takemoto[3], Tohru Miyoshi-Akiyama[3], Tomoko Sumitomo[1], Masanobu Nakata[4], Tadayoshi Ikebe[5], Tomoki Hanada[1], Takahiro Yamaguchi[6], Ryuji Kawahara[6], Rumi Okuno[7], Hitoshi Otsuka[8], Yuko Matsumoto[9], Yuji Terashima[10], Yu Kazawa[10], Noriko Nakanishi[11], Kaoru Uchida[12], Yumi Akiyama[13], Kaori Iwabuchi[14], Chikara Nakagawa[15], Kazunari Yamamoto[16], Victor Nizet[2,17], Shigetada Kawabata[1,*]

[1]Department of Oral and Molecular Microbiology, Osaka University Graduate School of Dentistry, Suita, Osaka, Japan
[2] Department of Pediatrics, University of California at San Diego School of Medicine, La Jolla, California 92093, USA
[3] Department of Infectious Diseases, Research Institute, National Center for Global Health and Medicine, Tokyo, Japan
[4]Department of Oral Microbiology, Kagoshima University Graduate School of Medical and Dental Sciences, Kagoshima, Japan
[5] Department of Bacteriology I, National Institute of Infectious Diseases, Tokyo, Japan
[6] Division of Microbiology, Osaka Institute of Public Health, Osaka city, Osaka, Japan
[7] Department of Microbiology, Tokyo Metropolitan Institute of Public Health, Tokyo, Japan
[8] Department of Public Health Sciences, Yamaguchi Prefectural Institute of Public Health and Environment Yamaguchi city, Yamaguchi, Japan
[9] Microbiological Testing and Research Division, Yokohama City Institute of Public Health, Yokohama, Kanagawa, Japan
[10] Department of Microbiology, Fukushima Prefectural Institute of Public Health, Fukushima city, Fukushima, Japan
[11] Department of Infectious Diseases, Kobe Institute of Health, Kobe, Hyogo, Japan
[12] Department of Bacteriology, Toyama Institute of Health, Imizu, Toyama, Japan
[13] Infectious disease research division, Hyogo Prefectural Institute of Public Health Science, Kakogawa, Hyogo, Japan
[14] Department of Health Science, Iwate Prefectural Research Institute for Environmental Sciences and Public Health, Morioka, Iwate, Japan
[15] Division of Microbiology, Kyoto City Institute of Health and Environmental Sciences, Kyoto city, Kyoto, Japan
[16] Niigata City Institute of Public Health and the Environment, Niigata city, Niigata, Japan
[17] Skaggs School of Pharmaceutical Sciences, University of California at San Diego, La Jolla, California 92093, USA

***Addresses for correspondence:** Yujiro Hirose, Department of Oral and Molecular Microbiology, Osaka University Graduate School of Dentistry, 1-8, Yamadaoka, Suita, Osaka 565-0871, Japan; Email: yujirohirose@dent.osaka-u.ac.jp
Shigetada Kawabata, Department of Oral and Molecular Microbiology, Osaka University Graduate School of Dentistry, 1-8, Yamadaoka, Suita, Osaka 565-0871, Japan; Email: kawabata@dent.osaka-u.ac.jp

SDC, Figure 1

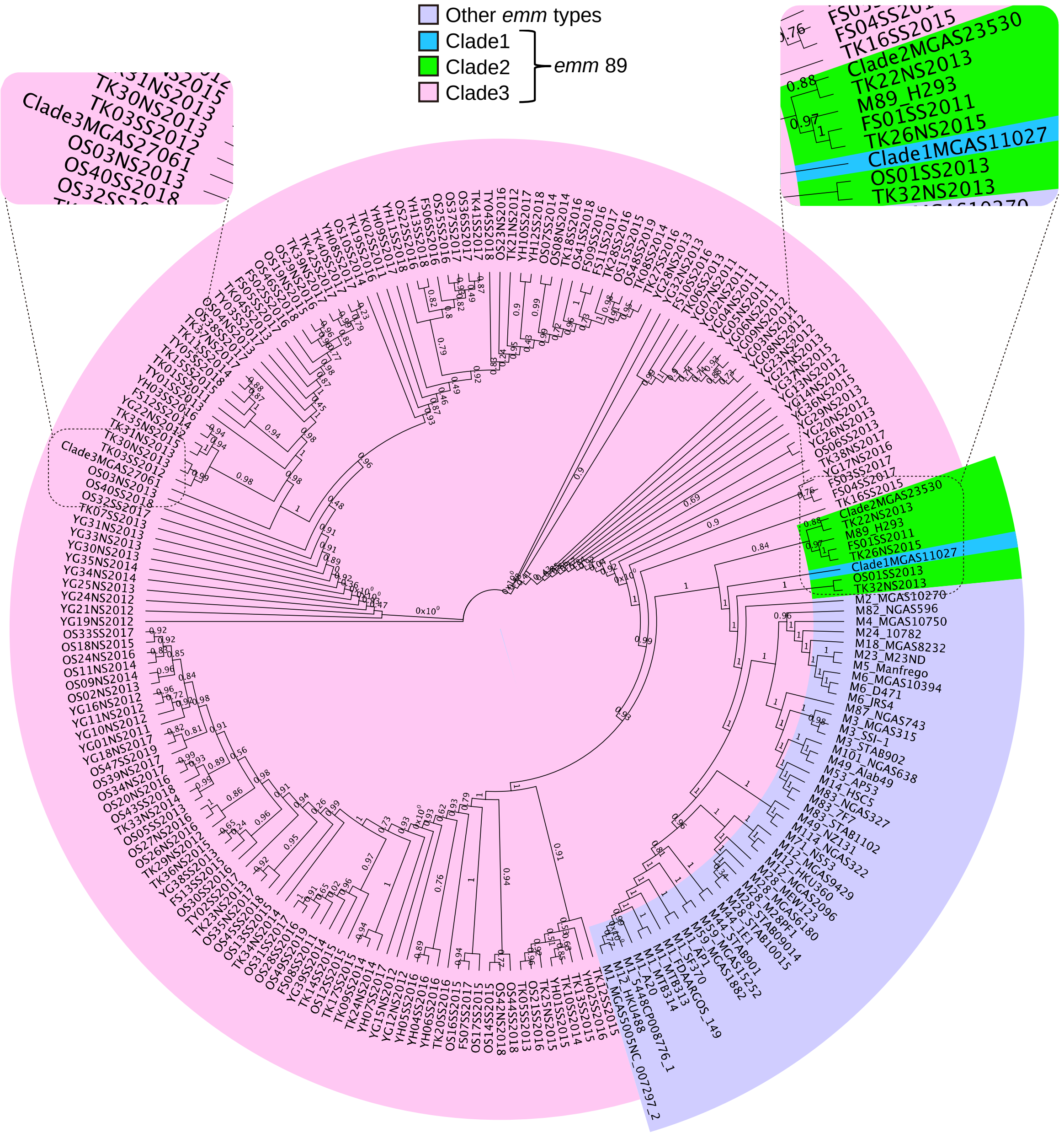

Supplementary Figure 1. Cladogram for Figure 1C. The cladogram is a branching diagram simply depicts sister group relationships and common ancestry. Branch supports represent bootstrap values. FS, regions containing Sapporo city, Iwate prefecture, Fukushima prefecture, Sendai city, and Niigata city; TY, Toyama prefecture; TK, Tokyo prefecture; YH, Yokohama OS, regions containing Shiga prefecture, Kyoto city, Osaka prefecture, and Hyogo prefecture. Yamaguchi prefecture. SS, isolates from STSS patients; NS, pharyngeal or asymptomatic isolates. 20xx indicates year of isolation.

SDC, Figure 2

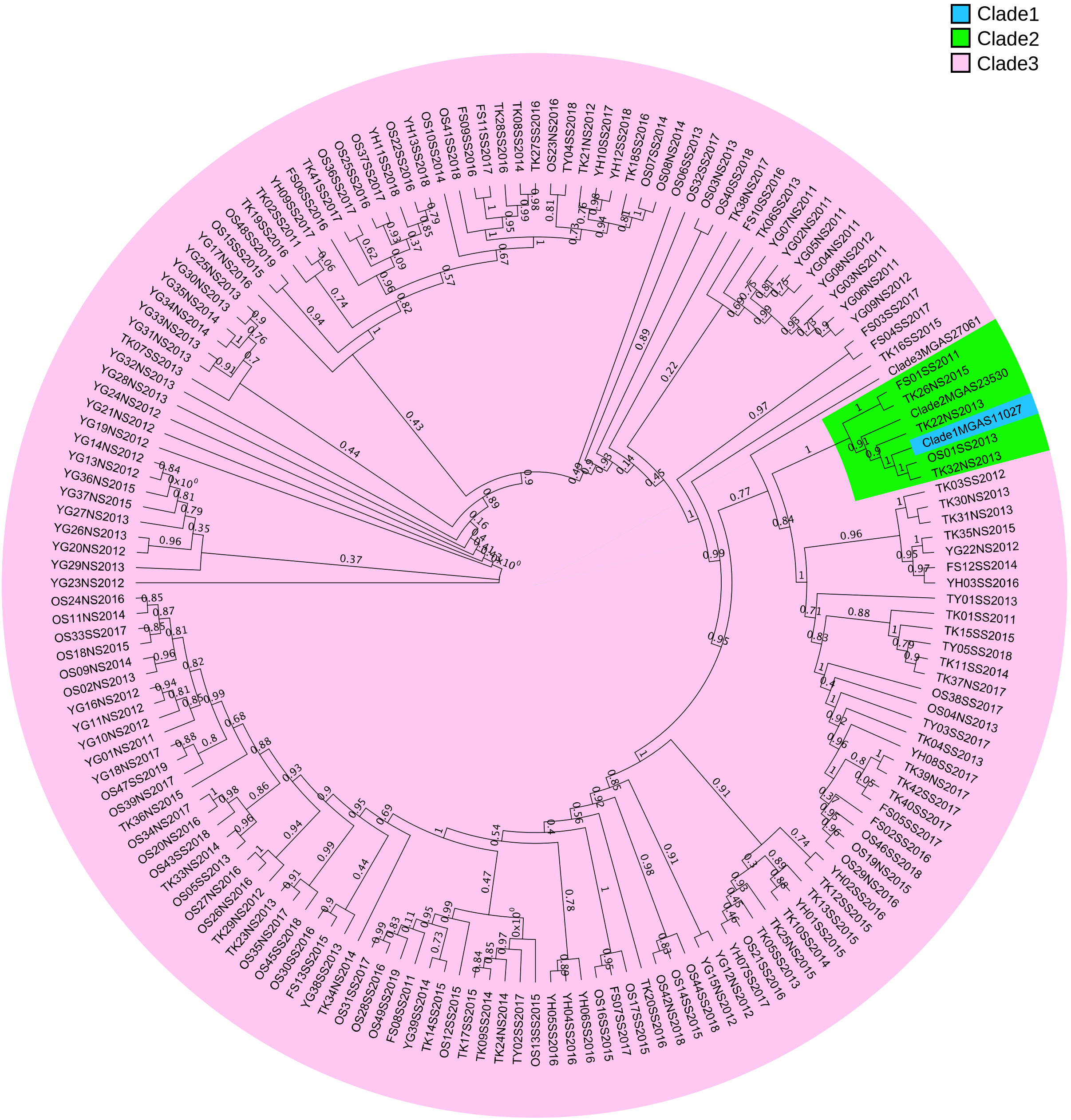

Supplementary Figure 2. Cladogram for Figure 2, Figure 3, and Figure S4. Branch supports represent bootstrap values.

SDC, Table 1

Supplementary Table 1. CMH test on invasive disease-associated factors

The regions applied to CHM test*	Factor	Detected number of isolates from invasive disease	Detected number of isolates from non-invasive disease	χ2 value**	P-value
TK, OS	Mutation in CovS	13 strains (Total 56 strains)	1 strain (Total 35 strains)	6.63	1.00E-02
TK, OS, YG	Absence of *hylP1* gene	25 strains (Total 58 strains)	10 strain (Total 72 strains)	7.79	5.30E-03
OS, YG	SagC (P223S)	2 strains (Total 33 strains)	9 strain (Total 55 strains)	4.33	3.75E-02
TK, OS	Ska (I17T)	15 strains (Total 56 strains)	3 strain (Total 35 strains)	5.71	1.69E-02

* To evaluate the impact of the deviation of the regions as a confounding factor, CMH test was conducted. However, our research does not contain the non-STSS isolates from FS, TY, and YH regions. In addition, CovS mutation in isolates from YG region, amino acid substitutions of SagC (P223S) in isolates from TK region, and amino acid substitutions of Ska (I17T) in isolates from YG region were not detected. Therefore, CMH tests were conducted using the area available for analysis.

**χ^2 values for evaluating association between STSS and protein effect.

SDC, Dataset 1. Strains and characteristics

No.	Strain	Country	Region	Year	Infection	Clade	CovS sequence alterations	HylA sequence	hylP gene	Hyaluronan-degrading activity	Reference
1	FS01	Japan	Iwate prefecture	2011	STSS	2	1 bp delete = stop at aa 35	inactive	-	-	13, 23
2	FS02	Japan	Iwate prefecture	2016	STSS	3	WT sequence	inactive	-	-	23
3	FS03	Japan	Iwate prefecture	2017	STSS	3	WT sequence	inactive	+	+	23
4	FS04	Japan	Iwate prefecture	2017	STSS	3	WT sequence	inactive	+	+	23
5	FS05	Japan	Iwate prefecture	2017	STSS	3	WT sequence	inactive	+	+	23
6	FS06	Japan	Fukushima prefecture	2016	STSS	3	WT sequence	inactive	+	+	23
7	FS07	Japan	Fukushima prefecture	2017	STSS	3	WT sequence	inactive	-	-	23
8	FS08	Japan	Sapporo city	2011	STSS	3	5 bp delete = stop at aa 37	inactive	-	-	13, 23
9	FS09	Japan	Sapporo city	2016	STSS	3	WT sequence	inactive	-	-	23
10	FS10	Japan	Sapporo city	2016	STSS	3	WT sequence	inactive	-	-	23
11	FS11	Japan	Sapporo city	2017	STSS	3	VLFF (23 aa to26 aa) to CSFFLHFL	inactive	-	-	23
12	FS12	Japan	Sendai city	2014	STSS	3	WT sequence	inactive	+	+	23
13	FS13	Japan	Niigata city	2015	STSS	3	A397T	inactive	-	-	23
14	OS01	Japan	Osaka city	2013	STSS	2	1 bp delete = stop at aa 35	inactive	-	-	23
15	OS02	Japan	Osaka prefecture	2013	non-STSS	3	WT sequence	inactive	+	+	23
16	OS03	Japan	Osaka prefecture	2013	non-STSS	3	WT sequence	inactive	+	+	23
17	OS04	Japan	Osaka prefecture	2013	non-STSS	3	WT sequence	inactive	+	+	23
18	OS05	Japan	Osaka city	2013	STSS	3	1 bp delete = stop at aa 35	inactive	+	+	23
19	OS06	Japan	Shiga prefecture	2013	STSS	3	WT sequence	inactive	+	+	23
20	OS07	Japan	Osaka city	2014	STSS	3	1 bp delete = stop at aa 35	inactive	-	-	23
21	OS08	Japan	Osaka prefecture	2014	non-STSS	3	WT sequence	inactive	-	-	23
22	OS09	Japan	Osaka prefecture	2014	non-STSS	3	WT sequence	inactive	+	+	23
23	OS10	Japan	Kobe city	2014	STSS	3	11 bp insert = stop at aa 39	inactive	+	+	23
24	OS11	Japan	Osaka prefecture	2014	non-STSS	3	WT sequence	inactive	+	+	23
25	OS12	Japan	Osaka prefecture	2015	STSS	3	D248Y	inactive	-	-	23

26	OS13	Japan	Osaka prefecture	2015	STSS	3	WT sequence	inactive	-	-	23
27	OS14	Japan	Osaka city	2015	STSS	3	WT sequence	inactive	+	+	23
28	OS15	Japan	Osaka city	2015	STSS	3	WT sequence	inactive	-	-	23
29	OS16	Japan	Osaka prefecture	2015	STSS	3	1 bp delete = stop at aa 35	inactive	-	-	23
30	OS17	Japan	Osaka prefecture	2015	STSS	3	WT sequence	inactive	-	-	23
31	OS18	Japan	Osaka prefecture	2015	non-STSS	3	WT sequence	inactive	+	+	23
32	OS19	Japan	Osaka prefecture	2015	non-STSS	3	WT sequence	inactive	+	+	23
33	OS20	Japan	Osaka prefecture	2016	non-STSS	3	WT sequence	inactive	-	-	23
34	OS21	Japan	Kobe city	2016	STSS	3	WT sequence	inactive	+	+	23
35	OS22	Japan	Kyoto city	2016	STSS	3	WT sequence	inactive	+	+	23
36	OS23	Japan	Osaka prefecture	2016	non-STSS	3	WT sequence	inactive	+	+	23
37	OS24	Japan	Osaka prefecture	2016	non-STSS	3	WT sequence	inactive	+	+	23
38	OS25	Japan	Hyogo prefecture	2016	STSS	3	WT sequence	inactive	+	+	23
39	OS26	Japan	Osaka prefecture	2016	non-STSS	3	WT sequence	inactive	+	+	23
40	OS27	Japan	Osaka prefecture	2016	non-STSS	3	WT sequence	inactive	+	+	23
41	OS28	Japan	Kobe city	2016	STSS	3	WT sequence	inactive	+	+	23
42	OS29	Japan	Osaka prefecture	2016	non-STSS	3	WT sequence	inactive	+	+	23
43	OS30	Japan	Hyogo prefecture	2016	STSS	3	WT sequence	inactive	-	-	23
44	OS31	Japan	Kobe city	2017	STSS	3	WT sequence	inactive	-	-	23
45	OS32	Japan	Kobe city	2017	STSS	3	WT sequence	inactive	+	+	23
46	OS33	Japan	Hyogo prefecture	2017	STSS	3	A38D	inactive	+	+	23
47	OS34	Japan	Osaka prefecture	2017	non-STSS	3	WT sequence	inactive	+	+	23
48	OS35	Japan	Osaka prefecture	2017	non-STSS	3	WT sequence	inactive	+	+	23
49	OS36	Japan	Amagasaki city	2017	STSS	3	WT sequence	inactive	-	-	23
50	OS37	Japan	Kobe city	2017	STSS	3	WT sequence	inactive	+	+	23
51	OS38	Japan	Hyogo prefecture	2017	STSS	3	WT sequence	inactive	+	+	23
52	OS39	Japan	Osaka prefecture	2017	non-STSS	3	WT sequence	inactive	+	+	23
53	OS40	Japan	Kyoto city	2018	STSS	3	1 bp delete = stop at aa 35	inactive	-	-	This study
54	OS41	Japan	Hyogo prefecture	2018	STSS	3	WT sequence	inactive	-	-	This study
55	OS42	Japan	Osaka prefecture	2018	non-STSS	3	WT sequence	inactive	+	+	This study
56	OS43	Japan	Osaka prefecture	2018	STSS	3	P16S	inactive	+	+	This study
57	OS44	Japan	Kobe city	2018	STSS	3	WT sequence	inactive	+	+	This study

58	OS45	Japan	Amagasaki city	2018	STSS	3	WT sequence	inactive	+	+	This study
59	OS46	Japan	Osaka city	2018	STSS	3	WT sequence	inactive	+	+	This study
60	OS47	Japan	Amagasaki city	2019	STSS	3	WT sequence	inactive	+	+	This study
61	OS48	Japan	Osaka prefecture	2019	STSS	3	A206T	inactive	-	-	This study
62	OS49	Japan	Osaka prefecture	2019	STSS	3	WT sequence	inactive	-	-	This study
63	TK01	Japan	Tokyo prefecture	2011	STSS	3	WT sequence	inactive	+	+	13, 23
64	TK02	Japan	Tokyo prefecture	2011	STSS	3	WT sequence	inactive	+	+	13, 23
65	TK03	Japan	Tokyo prefecture	2012	STSS	3	WT sequence	inactive	-	-	13, 23
66	TK04	Japan	Tokyo prefecture	2013	STSS	3	WT sequence	inactive	+	+	23
67	TK05	Japan	Tokyo prefecture	2013	STSS	3	WT sequence	inactive	+	+	23
68	TK06	Japan	Tokyo prefecture	2013	STSS	3	WT sequence	inactive	+	+	23
69	TK07	Japan	Tokyo prefecture	2013	STSS	3	11 bp insert = stop at aa 39	inactive	+	+	23
70	TK08	Japan	Tokyo prefecture	2014	STSS	3	WT sequence	inactive	-	-	23
71	TK09	Japan	Tokyo prefecture	2014	STSS	3	WT sequence	inactive	-	-	23
72	TK10	Japan	Tokyo prefecture	2014	STSS	3	WT sequence	inactive	+	+	23
73	TK11	Japan	Tokyo prefecture	2014	STSS	3	WT sequence	inactive	+	+	23
74	TK12	Japan	Tokyo prefecture	2015	STSS	3	WT sequence	inactive	+	+	23
75	TK13	Japan	Tokyo prefecture	2015	STSS	3	WT sequence	inactive	+	+	23
76	TK14	Japan	Tokyo prefecture	2015	STSS	3	WT sequence	inactive	-	-	23
77	TK15	Japan	Tokyo prefecture	2015	STSS	3	WT sequence	inactive	+	+	23
78	TK16	Japan	Tokyo prefecture	2015	STSS	3	WT sequence	inactive	-	-	23
79	TK17	Japan	Tokyo prefecture	2015	STSS	3	WT sequence	inactive	-	-	23
80	TK18	Japan	Tokyo prefecture	2016	STSS	3	Point mutation 283 stop	inactive	-	-	23
81	TK19	Japan	Tokyo prefecture	2016	STSS	3	1 bp delete = stop at aa 35	inactive	+	+	23
82	TK20	Japan	Tokyo prefecture	2016	STSS	3	WT sequence	inactive	+	+	23
83	TK21	Japan	Tokyo prefecture	2012	non-STSS	3	WT sequence	inactive	+	+	13, 23
84	TK22	Japan	Tokyo prefecture	2013	non-STSS	2	WT sequence	inactive	-	-	23
85	TK23	Japan	Tokyo prefecture	2013	non-STSS	3	WT sequence	inactive	+	+	23
86	TK24	Japan	Tokyo prefecture	2014	non-STSS	3	WT sequence	inactive	-	-	23
87	TK25	Japan	Tokyo prefecture	2015	non-STSS	3	WT sequence	inactive	+	+	23
88	TK26	Japan	Tokyo prefecture	2015	non-STSS	2	WT sequence	inactive	-	-	23
89	TK27	Japan	Tokyo prefecture	2016	STSS	3	WT sequence	inactive	-	-	23

90	TK28	Japan	Tokyo prefecture	2016	STSS	3	WT sequence	inactive	-	-	23
91	TK29	Japan	Tokyo prefecture	2012	non-STSS	3		inactive	+	+	13, 23
92	TK30	Japan	Tokyo prefecture	2013	non-STSS	3	WT sequence	inactive	+	+	23
93	TK31	Japan	Tokyo prefecture	2013	non-STSS	3	WT sequence	inactive	+	+	23
94	TK32	Japan	Tokyo prefecture	2013	non-STSS	2	WT sequence	inactive	+	+	23
95	TK33	Japan	Tokyo prefecture	2014	non-STSS	3	WT sequence	inactive	+	+	23
96	TK34	Japan	Tokyo prefecture	2014	non-STSS	3	WT sequence	inactive	+	+	23
97	TK35	Japan	Tokyo prefecture	2015	non-STSS	3	WT sequence	inactive	+	+	23
98	TK36	Japan	Tokyo prefecture	2015	non-STSS	3	G457V	inactive	-	-	23
99	TK37	Japan	Tokyo prefecture	2017	non-STSS	3	WT sequence	inactive	+	+	23
100	TK38	Japan	Tokyo prefecture	2017	non-STSS	3	WT sequence	inactive	+	+	23
101	TK39	Japan	Tokyo prefecture	2017	non-STSS	3	WT sequence	inactive	+	+	23
102	TK40	Japan	Tokyo prefecture	2017	STSS	3	WT sequence	inactive	+	+	23
103	TK41	Japan	Tokyo prefecture	2017	STSS	3	WT sequence	inactive	-	-	23
104	TK42	Japan	Tokyo prefecture	2017	STSS	3	1 bp delete = stop at aa 35	inactive	+	+	23
105	TY01	Japan	Toyama prefecture	2013	STSS	3	WT sequence	inactive	-	-	23
106	TY02	Japan	Toyama prefecture	2017	STSS	3	WT sequence	inactive	-	-	23
107	TY03	Japan	Toyama prefecture	2017	STSS	3	WT sequence	inactive	+	+	23
108	TY04	Japan	Toyama prefecture	2018	STSS	3	WT sequence	inactive	+	+	This study
109	TY05	Japan	Toyama prefecture	2018	STSS	3	WT sequence	inactive	+	+	This study
110	YG01	Japan	Yamaguchi prefecture	2011	non-STSS	3	WT sequence	inactive	+	+	13, 23
111	YG02	Japan	Yamaguchi prefecture	2011	non-STSS	3	WT sequence	inactive	+	+	13, 23
112	YG03	Japan	Yamaguchi prefecture	2011	non-STSS	3	WT sequence	inactive	+	+	13, 23
113	YG04	Japan	Yamaguchi prefecture	2011	non-STSS	3	WT sequence	inactive	+	+	13, 23
114	YG05	Japan	Yamaguchi prefecture	2011	non-STSS	3	WT sequence	inactive	+	+	13, 23
115	YG06	Japan	Yamaguchi prefecture	2011	non-STSS	3	WT sequence	inactive	+	+	13, 23

116	YG07	Japan	Yamaguchi prefecture	2011	non-STSS	3	WT sequence	inactive	+	+	13, 23
117	YG08	Japan	Yamaguchi prefecture	2012	non-STSS	3	WT sequence	inactive	+	+	13, 23
118	YG09	Japan	Yamaguchi prefecture	2012	non-STSS	3	WT sequence	inactive	+	+	13, 23
119	YG10	Japan	Yamaguchi prefecture	2012	non-STSS	3	WT sequence	inactive	+	+	13, 23
120	YG11	Japan	Yamaguchi prefecture	2012	non-STSS	3	WT sequence	inactive	+	+	13, 23
121	YG12	Japan	Yamaguchi prefecture	2012	non-STSS	3	WT sequence	inactive	+	+	13, 23
122	YG13	Japan	Yamaguchi prefecture	2012	non-STSS	3	WT sequence	inactive	+	+	13, 23
123	YG14	Japan	Yamaguchi prefecture	2012	non-STSS	3	WT sequence	inactive	+	+	13, 23
124	YG15	Japan	Yamaguchi prefecture	2012	non-STSS	3	WT sequence	inactive	+	+	13, 23
125	YG16	Japan	Yamaguchi prefecture	2012	non-STSS	3	WT sequence	inactive	+	+	13, 23
126	YG17	Japan	Yamaguchi prefecture	2016	non-STSS	3	WT sequence	inactive	+	+	23
127	YG18	Japan	Yamaguchi prefecture	2017	non-STSS	3	WT sequence	inactive	+	+	23
128	YG19	Japan	Yamaguchi prefecture	2012	non-STSS	3	WT sequence	inactive	+	+	13, 23
129	YG20	Japan	Yamaguchi prefecture	2012	non-STSS	3	WT sequence	inactive	+	+	13, 23
130	YG21	Japan	Yamaguchi prefecture	2012	non-STSS	3	WT sequence	inactive	+	+	13, 23
131	YG22	Japan	Yamaguchi prefecture	2012	non-STSS	3	WT sequence	inactive	-	-	13, 23

132	YG23	Japan	Yamaguchi prefecture	2012	non-STSS	3	WT sequence	inactive	+	+	13, 23
133	YG24	Japan	Yamaguchi prefecture	2012	non-STSS	3	WT sequence	inactive	+	+	13, 23
134	YG25	Japan	Yamaguchi prefecture	2013	non-STSS	3	WT sequence	inactive	+	+	23
135	YG26	Japan	Yamaguchi prefecture	2013	non-STSS	3	WT sequence	inactive	+	+	23
136	YG27	Japan	Yamaguchi prefecture	2013	non-STSS	3	WT sequence	inactive	+	+	23
137	YG28	Japan	Yamaguchi prefecture	2013	non-STSS	3	WT sequence	inactive	+	+	23
138	YG29	Japan	Yamaguchi prefecture	2013	non-STSS	3	WT sequence	inactive	+	+	23
139	YG30	Japan	Yamaguchi prefecture	2013	non-STSS	3	WT sequence	inactive	+	+	23
140	YG31	Japan	Yamaguchi prefecture	2013	non-STSS	3	WT sequence	inactive	+	+	23
141	YG32	Japan	Yamaguchi prefecture	2013	non-STSS	3	WT sequence	inactive	-	-	23
142	YG33	Japan	Yamaguchi prefecture	2013	non-STSS	3	WT sequence	inactive	+	+	23
143	YG34	Japan	Yamaguchi prefecture	2014	non-STSS	3	WT sequence	inactive	-	-	23
144	YG35	Japan	Yamaguchi prefecture	2014	non-STSS	3	WT sequence	inactive	-	-	23
145	YG36	Japan	Yamaguchi prefecture	2015	non-STSS	3	WT sequence	inactive	+	+	23
146	YG37	Japan	Yamaguchi prefecture	2015	non-STSS	3	WT sequence	inactive	+	+	23
147	YG38	Japan	Yamaguchi prefecture	2013	STSS	3	WT sequence	inactive	-	-	23

148	YG39	Japan	Yamaguchi prefecture	2014	STSS	3	WT sequence	inactive	+	+	23
149	YH01	Japan	Yokohama city	2015	STSS	3	WT sequence	inactive	+	+	23
150	YH02	Japan	Yokohama city	2016	STSS	3	WT sequence	inactive	+	+	23
151	YH03	Japan	Yokohama city	2016	STSS	3	WT sequence	inactive	+	+	23
152	YH04	Japan	Yokohama city	2016	STSS	3	WT sequence	inactive	+	+	23
153	YH05	Japan	Yokohama city	2016	STSS	3	WT sequence	inactive	+	+	23
154	YH06	Japan	Yokohama city	2016	STSS	3	WT sequence	inactive	+	+	23
155	YH07	Japan	Yokohama city	2017	STSS	3	WT sequence	inactive	+	+	23
156	YH08	Japan	Yokohama city	2017	STSS	3	WT sequence	inactive	+	+	23
157	YH09	Japan	Yokohama city	2017	STSS	3	R320C, H341Y	inactive	+	+	23
158	YH10	Japan	Yokohama city	2017	STSS	3	WT sequence	inactive	+	+	23
159	YH11	Japan	Yokohama city	2018	STSS	3	1 bp insert = stop at aa 39	inactive	+	+	This study
160	YH12	Japan	Yokohama city	2018	STSS	3	WT sequence	inactive	+	+	This study
161	YH13	Japan	Yokohama city	2018	STSS	3	V277A	inactive	+	+	This study

SDC, Dataset 2. *Streptococcus pyogenes* complete genome sequences (as of 11/1/2019)

No.	M-type	Strain	NCBI Accession	Length (nts)	%G+C	CDSs	Collection year	Registration year
1	89	MGAS11027	CP013838	1786874	38.55	1682	2002	2016
2	89	MGAS23530	CP013839	1709394	38.51	1593	1997	2016
3	89	MGAS27061	CP013840	1741348	38.52	1626	2008	2016
4	1	5448	CP008776	1829516	38.5	1723	1994	2015
5	1	A20	CP003901.1	1837281	38.54	1828		2012
6	1	AP1	CP007537	1908294	38.47	1836		2015
7	1	FDAARGOS_149	CP014027	1839641	38.54	1745	2014	2013
8	1	MGAS5005	CP000017	1838554	38.5	1865		2005
9	1	MTB313	AP014572	1745332	38.52	1758	2011	2015
10	1	MTB314	AP014585	1744827	38.51	1658	2011	2015
11	1	SF370	AE004092	1852441	38.5	1697		2010
12	2	MGAS10270	CP000260	1928252	38.4	1986		2006
13	3	MGAS315	AE014074	1900521	38.6	1865		2003
14	3	SSI-1	BA000034	1894275	38.6	1861		2004
15	3	STAB902	CP007041.1	1892124	38.5	1809		2014
16	4	MGAS10750	CP000262	1937111	38.3	1979		2006
17	5	Manfredo	AM295007	1841271	38.6	1745	1950's	2007
18	6	D471	CP011415	1811968	38.63	1671	1971	2015
19	6	JRS4	CP011414	1811968	38.63	1671	1971	2015
20	6	MGAS10394	CP000003	1899877	38.7	1886		2004
21	12	HKU360	CP009612.1	1944537	38.46	1846		2014
22	12	HKU488	CP012045	1943415	38.45	1882	2012	2015
23	12	MGAS2096	CP000261	1860355	38.7	1898		2006
24	12	MGAS9429	CP000259	1836467	38.5	1877		2006
25	14	HSC5	CP006366.1	1818351	38.5	1744		2013
26	18	MGAS8232	AE009949	1895017	38.5	1839		2003
27	23	M23ND	CP008695.1	1846477	38.61	1842	2012	2014
28	24	10782	GL397225	1,838,678	38.4	1943		2010

29	28	M28PF1	CP011535	1896976	38.35	1765	2009	2015
30	28	MEW123	CP014139	1878699	38.29	1738	2012	2016
31	28	MGAS6180	CP000056	1897573	38.4	1894	1998	2005
32	28	STAB09014	CP011069	1862487	38.36	1707	2009	2015
33	28	STAB10015	CP011068	1950454	38.25	1810	2010	2015
34	44	1E1	CP007241.1	1796152	38.48	1652	2009	2014
35	44	STAB901	CP007024.1	1795609	38.5	1358		2014
36	49	Alab49	CP003068.1	1827308	38.58	1773	1986	2011
37	49	NZ131	CP000829	1815785	38.6	1700		2008
38	53	AP53	CP013672	1860554	38.56	1840	1967	2016
39	59	MGAS15252	CP003116.1	1750832	38.5	1662		2012
40	59	MGAS1882	CP003121.1	1781029	38		early 1960	2012
41	71	NS53	CP015238	1765123	38.42	1630	1990	2016
42	82	NGAS596	CP007561	1791306	38.53	1626		2015
43	83	7F7	CP007240.1	1709790	38.6	1550	2011	2014
44	83	NGAS327	CP007562	1702054	38.62	1546		2015
45	83	STAB1102	CP007023.1	1709442	38.6	1582		2014
46	87	NGAS743	CP007560	1915554	38.5	1807		2015
47	89	H293	HG316453	1726248	38.55	1601		2015
48	101	NGAS638	CP010450	1791401	38.56	1654		2015
49	114	NGAS322	CP010449	1950469	38.32	1812		2015

Indicates the 23 serotypes types and 49 strains used in this study.
